# Perspectives on a US–Mexico Border Community’s Diabetes and “Health-Care” Access Mobilization Efforts and Comparative Analysis of Community Health Needs over 12 Years

**DOI:** 10.3389/fpubh.2017.00152

**Published:** 2017-07-10

**Authors:** Cecilia Ballesteros Rosales, Jill Eileen Guernsey de Zapien, Jean Chang, Maia Ingram, Maria L. Fernandez, Scott C. Carvajal, Lisa K Staten

**Affiliations:** ^1^Mel and Enid Zuckerman College of Public Health, University of Arizona, Tucson, AZ, United States; ^2^Resident Promotora of Douglas Arizona, Douglas, AZ, United States; ^3^Richard M. Fairbanks School of Public Health, Indiana University–Purdue University Indianapolis, Indianapolis, IN, United States

**Keywords:** community health, community engagement, border health survey, border health, community-based participatory research

## Abstract

This paper describes a community coalition–university partnership to address health needs in an underserved US–Mexico border, community. For approximately 15 years, this coalition engaged in community-based participatory research with community organizations, state/local health departments, and the state’s only accredited college of public health. Notable efforts include the systematic collection of health-relevant data 12 years apart and data that spawned numerous health promotion activities. The latter includes specific evidence-based chronic disease-preventive interventions, including one that is now disseminated and replicated in Latino communities in the US and Mexico, and policy-level changes. Survey data to evaluate changes in a range of health problems and needs, with a specific focus on those related to diabetes and access to health-care issues—identified early on in the coalition as critical health problems affecting the community—are presented. Next steps for this community and lessons learned that may be applicable to other communities are discussed.

## Introduction

In the mid-1990s, a core partnership emerged committed to understanding and acting upon health problems found among a highly underserved community located along the US–Mexico border in Southeast Arizona. The partnership started with a group of local community residents in the municipality of Douglas, Arizona, and allies at the nearest public university (University of Arizona) and state of Arizona’s Department of Health Services (ADHS). This group of grassroots health activists, researchers, and policymakers had anecdotal evidence for a major health problem in the area associated with inadequate access to the health care as well as diabetes and diabetes-related complications and charged that systematic data collection was needed to better grasp the scope and depth of the problem (1).

Consequently, the partnership planned and executed the first Douglas Community Health survey in 1997–1998—a mixed methods random proportional household survey. The survey was conducted with contributed resources from many of the organizations participating in a community coalition. Researchers collected information on a diverse range of health problems and solicited responses to health utilization questions. Participants also responded to open-ended questions to discuss and reflect on community health needs. Trained local residents and promotoras (community health workers) administered the survey. The survey data collected applied high rigor in terms of public health surveillance metrics and included a sample population approaching 1,000 residents.

The data collected were widely shared with all members of the partnership. For example, these data supported the growth of a local health center, the strengthening of local non-profit organizations, and the direction of local and state health resources. In addition, these data helped launch federally funded research to develop new interventions on diabetes and promote and monitor community health changes from a multi-level and social ecological perspective (e.g., consider individual-behavioral, social environment, and policy change). Two central themes permeated all these activities; community activists and promotoras were highly involved in community assessment, engagement, and intervention development and execution, and the community partnership was central in guiding all research activities.

One specific effort/outcome of the partnership and the community data was the identification of need for improved chronic disease prevention and management curriculums and programs. As a result, a series of interventions were developed and tested. One of these interventions is *Pasos Adelante* (“Steps Forward”). Funded through core research sponsored by the United States Centers for Disease Control and Prevention’s National Center for Chronic Disease Prevention and Health Promotion, Pasos Adelante was designed to prevent diabetes and reduce health-related complications from chronic disease in high-risk Hispanic populations. This intervention has a strong evidence base, including work conducted in Douglas Arizona (2–4) and, further, elements of the program believed central to its effectiveness (e.g., walking groups) were implemented even after the federal funding ended. Further, this intervention is being promoted as an evidence-based intervention by the Centers for Disease Control and Prevention (5). The intervention has been introduced to other predominantly Latino communities in other states. Furthermore, adaptation for a Mexican national population has shown promising results in Mexico (6, 7). The need for these and related effective interventions are still critical. Diabetes among Hispanics, the largest ethnic and racial majority group in the US, remain nearly double when compared to White non-Hispanics. Further, among Hispanics, Mexican Americans who compose the largest sub-group (65%), have one of the largest rates. Moreover, Mexico has the most alarming rates of diabetes and major gaps in its health infrastructure to reduce its population-level mortality and quality-of-life toll.

While examples, like Pasos Adelante as well as others, illustrate how the partnership addressed health needs through improved individual-, group-, and family-based health promotion strategies and created translatable interventions, the partnership also directed attention to community and policy changes. One key effort was around another key theme from the initial survey data, the need for improved access to care in the community. The partnership and data helped contribute to the major growth of a federally qualified health center in the area, with a main clinic close to Douglas residents and with large-scale education on the eligibility of potential services. Other efforts included policy and education efforts with the K–12 system to promote physical activity and improve diet. In addition, the partnership, as well as many other stakeholders statewide, helped promote a successful effort statewide that expanded Medicaid to 133% of the federal poverty level. Analysis suggests these collective policies and systems change reduced the gaps in health insurance in the Douglas community, particularly pronounced among residents with lower education and income levels (8). The above said, in the US as a whole, health coverage and health utilization disparities are greater for Mexican-origin persons than for other groups, and these patterns are consistent in those eligible for Medicaid services (among those who qualify for legal residence status or citizenship, income levels, and family size). Other data also show that within the US–Mexico border region, these disparities are further exacerbated (9).

### A Snapshot of Douglas Arizona and Data from 12 Years

Douglas, Arizona, is recognized by the United States Health Services Administration as rural and underserved (10) by health professionals. US Census data also show comparatively low educational attainment and high poverty (11). However, two detailed community health surveys, the previously described survey as well as a similar one completed in 2011, afford a unique opportunity to explore changes in this community in terms of chronic disease prevention and control as well as health-care coverage and utilization. Further, the more recent survey also provides an opportunity to examine identified health problems faced by the community. The remaining focus of this paper involves contrasting the data from the two surveys and illuminating future community responsive public health promotion needs.

## Materials and Methods

Both surveys (1996 and 2010) grew from concerns and interest on the part of the community coalition and partnership with Arizona Prevention Research Center at the University of Arizona Mel & Enid Zuckerman College of Public Health. The methods related to data collection and our community-based participatory research (12) approaches is reported in previously published articles (1, 8, 13). However, it is important to highlight that both surveys were community driven, including participation in the design, implementation, and interpretation of the findings. Even though an 11-year period transpired between the two surveys, the same institutions and many of the same individuals participated in the design, implementation, and analysis of both surveys in 1996 and 2010. For example, one of four interviewers participating in the collection of data in both surveys is a co-author on this paper. The study participants were drawn from a randomized cross-sectional community survey and are representative of the municipality of Douglas, Arizona. Strata of census blocks were identified by ethnicity and socioeconomic status to reflect socio-demographic distribution. Occupied housing units were then randomly chosen from the selected census areas.

## Results

Reflected in the data presented in Table 1 are shifts in study participants’ age, preferred language, and education. Note the older population surveyed in 2010 (53 years of age) compared to the 1996 cohort (45 years of age). This is in part related to demographic trends (about 2 years older in median US census age in that community in 2010 than 2000), but may also reflect the comfort of the respondents with the maturity of the interviewers—all interviewers were 50 years of age or older. The preferred language in both surveys was Spanish. We also find a significant difference in educational attainment; more high school and less college in second survey, but less high school is constant.

**Table 1:** Demographics and health service utilization in Douglas, Arizona, 1988 and 2010.

Variables	1997–1998 (*N* = 915)	2010–2011 (*N* = 708)
**Age (SD)**	44.77 (±15.3)	52.77 (±19.0)
**Gender, *n* (%)**		
Male	307 (33.6)	264 (37.3)
Female	608 (66.4)	444 (62.7)
**Hispanic, *n* (%)**		
Yes	852 (93.1)	649 (91.7)
No	63 (6.9)	59 (8.3)
**Nativity, *n* (%)**		
US-born	366 (40.0)	303 (42.8)
Foreign-born	549 (60.0)	405 (57.2)
**High school education or above, *n* (%)**	458 (50.1)	406 (57.3)
**Saw a health provider in the last year, *n* (%)**	671 (73.3)	628 (88.7)
**Usual source of care, *n* (%)**		
None	69 (7.5)	24 (3.4)
Private doctor	272 (29.7)	194 (27.4)
Health center (public)	511 (55.8)	479 (67.7)
Other	63 (6.9)	40 (5.7)
**Emergency department visit, last 6 months, *n* (%)**	109 (11.9)	88 (12.4)
**Insurance coverage (%)**		
Uninsured	33 (3.6)	18 (2.5)
Medicaid	23 (2.5)	41 (5.7)
Medicare	7 (0.7)	28 (3.9)
Private	11 (1.2)	26 (3.6)
**Tested in the last 12 months (%)**		
Blood pressure	76 (8.3)	88 (12.4)
Urine	53 (5.7)	78 (11.0)
Vision	45 (4.9)	48 (6.7)

In terms of access to health care, Table 1 shows an increase in Medicaid, Medicare, and private insurance enrollment. Also in Table 1, we see higher utilization rates in screening behaviors with the exception of vision screening. These higher rates indicate an overall increase in access to care with the exception of vision screening and care.

In addition, our study shows that, in Douglas, the age-adjusted rate of diabetes prevalence increased from 12.5 to 13.8% (change of 1.3%) in the 12 years between the surveys. In the state of Arizona, the change was 2.8 to 7.1% (4.3%) and national age-adjusted rate increased from 4.9 to 7.9% (3.0%). The unadjusted data show that, in Douglas, the prevalence increased from 13.2 to 19.6% (6.4%) in the 12 years between the surveys. In the state of Arizona, the change was 2.8 to 9.0% (6.2%) and national unadjusted rate increased from 5.4 to 8.7% (3.3%).

Further, as Table 2 illustrates, the 18- to 24-year-old group in Douglas had no change. Moreover, no change was observed in Arizona prevalence and in national prevalence in this age group. The 25- to 34-year-old group’s diabetes prevalence in Douglas decreased from 7 to 2.5% (4.5%), decreased in Arizona prevalence from 0.6 to 0.5% (0.1%), and decreased in national prevalence from 1 to 2.2% (1.2%). The prevalence of diabetes in the 35- to 44-year-old group decreased from 11.1 to 6.1% (5.0%), increased in Arizona prevalence from 2.1 to 4.1% (2.0%), and increased in national prevalence from 2.2 to 4.3% (2.1%). The 45- to 54-year-old group in Douglas increased in their prevalence from 12.7 to 16.1% (3.4%), increased in Arizona from 4.1to 9.3% (5.2%), and increased nationally from 5.2 to 8.4% (3.2%). In Douglas, the 55- to 64-year-old group’s diabetes prevalence increased from 23.1 to 32.3% (9.2%), Arizona prevalence increased from 5.9 to 15.7% (9.8%), and national prevalence increased from 10.4 to 15.7% (5.3%). Finally, the 65+ years old group’s diabetes prevalence in Douglas increased from 25.2 to 34.0% (8.8%), Arizona prevalence increased from 5.1 to 16.9% (11.8%), and national prevalence increased from 12.5 to 19.5% (7.0%).

**Table 2:** Diabetes prevalence, control, and related health service utilization in Douglas Arizona, 1998 and 2010.

Variables	1997–1998 (*N* = 915)		2010–2011 (*N* = 708)	
Diabetes, cases (total prevalence %)	121 (13.2%)		139 (19.6%)	
Age 18–24	0 (0%)		0 (0%)	
Age 25–34	12 (7.0%)		2 (2.5%)	
Age 35–44	25 (11.1%)		6 (6.1%)	
Age 45–54	20 (12.7%)		20 (16.1%)	
Age 55–64	30 (23.1%)		40 (32.3%)	
Age 65+	34 (25.2%)		71 (34.0%)	
Diabetes, age-adjusted prevalence, %	12.5%		13.8%	
Yearly glucose check-up, *n* (%)***	476 (52.0%)		574 (81.0%)	
Diabetes control, *n* (%)^a^***	Controlled	Uncontrolled	Controlled	Uncontrolled
	62 (51.2%)	59 (48.8%)	85 (61.2%)	54 (38.8%)
Diabetes medical treatment, *n* (%)^b^***	Yes	No	Yes	No
	673 (73.5%)	242 (26.5%)	648 (91.5%)	60 (8.5%)
Yearly glucose check-up, *n* (%)***	476 (52.0%)		574 (81.0%)	
Diabetes control, *n* (%)^a^***	Controlled	Uncontrolled	Controlled	Uncontrolled
	62 (51.2%)	59 (48.8%)	85 (61.2%)	54 (38.8%)
Diabetes medical treatment, *n* (%)^b^***	Yes	No	Yes	No
	673 (73.5%)	242 (26.5%)	648 (91.5%)	60 (8.5%)

*^a^Defined by one-time random finger stick blood glucose test level using American Diabetes Association guideline*.

*^b^Voluntary medical treatment compliance for diabetes*.

***p < 0.001.

*χ^2^ value for yearly glucose check-up: 30.7, χ^2^ value for diabetes control: 10.4, χ^2^ value for diabetes medical treatment: 8.4*.

## Discussion

Following the initial study in 1996, every effort was expended to collaborate in advocating for resources from the local, state, and federal levels to respond to what was reportedly a higher prevalence of diabetes and poor rates of control in this small community compared to the state and national rates (14). There was a strong focus of the community to increase access to care including expansion of community health centers, educational programs to improve health behaviors as well as promote social support, and policy initiatives (1). The comparative data in the second survey revealed a dramatic shift in access to care within the community—most plausibly in response to actions at the local, state, and national level. During that period, Arizona increased its eligibility guidelines for Medicaid from 33% of the federal guidelines to 100% of the guidelines. Additional programs for the uninsured were developed with tobacco tax monies, and both the federally qualified community health center and a rural health clinic expanded services in the community. While the data also show an increase in Medicare coverage in this population in part a reflection of the older sample in the second survey, the increases were far greater than that expected by age differences alone. Another factor that may have contributed to increased care was outreach efforts related to the national/state policy changes as well as from local health service providers and grassroots and community coaltion efforts.

The findings also showed dramatic increases in regular screenings for diabetes and blood pressure; the percent of diabetics in regular care and the percent of diabetics whose glucose was under control a reflection of the overall shift in access to care and heightened awareness about diabetes by health providers and community members. The health behaviors and vital data measured at the community level, on the other hand, did not suggest population-level increases in preventive behavior based on the two surveys. However, we do see a decrease in physical activity and smoking. Additionally, if we look at the information in the second survey, we see 63% of the population meets the Centers for Disease Control and Prevention recommended criteria of 150 min of moderate or 75 min of vigorous activity per week as compared to US. and Arizona data. It should be noted, however, that both surveys were cross-sectional, and exposures to multiple types of factors were occurring. Thus while there are dramatic shifts in a number of health problems recognized by researchers, policymakers, and community members in the initial survey—namely diabetes and access to care—there is no way to precisely identify which health promotion strategies or which secular changes are most prominent in the improved health outcomes observed over a decade later.

## Conclusion

As described by population health scholars, the largest shifts—community health improvements—may require coordinated action at the local level and larger policy levels, with actions that span health and non-health sectors (15). This paper describes two population health improvements, specifically improved health-care access and improved diabetes control that dramatically improved within an underserved and impoverished community at the US–Mexico border across a span of 12 years. These improvements are likely to combined efforts of policymakers, researchers, and community members who identified specific and critical health problems—and some of these efforts were in direct responses to the data provided in an initial survey and shared across sectors.

As previously noted, the administration of these two surveys are the result of a strong partnership between the Arizona Prevention Research Center at the University of Arizona Mel & Enid Zuckerman College of Public Health, state and local health departments, and a local community coalition. In presenting the data to the community, there was a clear impact on the burden of diabetes with a strong emphasis on increasing access and quality of care, identification of diabetics, and control of diabetes.

Perhaps one of the important lessons learned when communities face an overwhelming burden of chronic disease is that the first efforts to address this issue should focus on individuals with diabetes and insure they have access and quality of care to control their disease. With significant movement in this arena, public health efforts obviously must continue to address both prevention and control of diabetes in the community. While there have been numerous individual organizational interventions focusing on prevention as well as specific policy interventions, there is a real need to develop a comprehensive prevention plan for the community. Now with the Affordable Care Act, there is an important opportunity both for continuing to increase access to quality care and to build stronger prevention programs in the community.

## Ethics Statement

This study was carried out in accordance with the recommendations of the University of Arizona Institutional Review Board with written informed consent from all subjects. All subjects gave informed consent in accordance with the Declaration of Helsinki. The protocol was approved by the University of Arizona Institutional Review Board.

## Author Contributions

CR was the primary writer of the article and along with JZ and MF was involved in the planning, implementation, and community engagement involved with both surveys described. JC was the principal data analyst. MI, LS, and SC were involved in the design and execution of second survey (2010) and involved closely with many of the community interventions described and future research directions identified. All authors contributed to, reviewed, and approved the final version of the manuscript and are accountable for the work presented.

## Conflict of Interest Statement

The authors declare that the research was conducted in the absence of any commercial or financial relationships that could be construed as a potential conflict of interest.
